# Iopromide- and gadopentetic acid-derived preparates used in MR arthrography may be harmful to chondrocytes

**DOI:** 10.1186/s13018-017-0600-5

**Published:** 2017-06-26

**Authors:** Kadir Oznam, Duygu Yasar Sirin, Ibrahim Yilmaz, Yasin Emre Kaya, Mehmet Isyar, Seyit Ali Gumustas, Hanefi Ozbek, Semih Akkaya, Arda Kayhan, Mahir Mahirogullari

**Affiliations:** 1 Department of Orthopaedic and Traumatology, Istanbul Medipol University School of Medicine, 34214 Istanbul, Turkey; 2 Department of Molecular Biology and Genetic, Namik Kemal University Faculty of Arts and Sciences, 59100 Tekirdag, Turkey; 3 Department of Medical Pharmacology, Istanbul Medipol University School of Medicine, 34810 Istanbul, Turkey; 4 Republic of Turkey, Ministry of Health, Department of Orthopaedic and Traumatology, Corlu State Hospital, 59100 Tekirdag, Turkey; 5 Department of Orthopaedic and Traumatology, Acibadem Hospitals Group, 34180 Istanbul, Turkey; 6 Republic of Turkey, Ministry of Health, Dr. Lutfi Kirdar Research and Training Hospital, 34890 Istanbul, Turkey; 7 Department of Orthopaedic and Traumatology, Denizli Private Surgery Hospital, 20070 Denizli, Turkey; 8 Department of Radiology, Istanbul Kanuni Sultan Suleyman Training and Research Hospital, 34303 Istanbul, Turkey; 9 Department of Orthopaedic and Traumatology, Memorial Health Group, 34384 Istanbul, Turkey

**Keywords:** MR-arthrography, Gadopentetic acid, Iopromide, Chondrotoxicity, Primary cell culture, Stage-specific embryonic antigen-1

## Abstract

**Background:**

Magnetic resonance arthrography, a procedure through which contrast agents containing gadolinium and/or iopromide are administered intra-articularly, has become a useful tool in musculoskeletal diagnosis. Nevertheless, despite being considered safe for systemic use, certain tissue toxicities have been identified for both drugs. In this study, the effects of short-term exposure of human primary chondrocyte cell cultures to gadolinium and/or iopromide contrast agents were examined by assaying for stage-specific embryonic antigen-1 (SSEA-1) protein expression (a chondrogenic differentiation marker), cell viability, toxicity, and proliferation.

**Methods:**

Human articular chondrocytes were grown in monolayer culture and were exposed to iopromide and/or gadolinium diethylenetriamine-pentaacetate (Gd-DPT) for 2 and 6 h. Cell cultures with no drug exposure were used as the control group. Cell differentiation status was assessed according to SSEA-1 protein expression. Contrast agent effects on cell viability and proliferation were analyzed using MTT analysis. Further, changes in cell morphology in relation to the control group were evaluated using inverted light microscopy, environmental scanning electron microscopy (ESEM), and 3-tesla magnetic resonance imaging. The obtained data were statistically compared.

**Results:**

When compared with the control group, both SSEA-1 protein expression and cell proliferation were lowest in the Gd-DPT group (*P* = 0.000). There was a statistically significant correlation between SSEA-1 expression and MTT results (rho = 0.351; *P* = 0.003).

**Conclusions:**

Nevertheless, the data obtained from in vitro experiments may not directly correspond to clinical applications. However, the mere fact that a drug used solely for diagnostic purposes may repress chondrocyte cell proliferation should be carefully considered by clinicians.

## Background

Arthrographic contrast agents are commonly used to determine free intra-articular objects and to diagnose shoulder labroligament abnormalities, rotator cuff tendon damage, partial and full-thickness elbow tears, hip joint labral tears, residual or recurrent knee tears following meniscectomy, triangle fibrocartilage and ligamentary damage in carpus, and impingement syndrome in the ankle [1, 2].

Arthography induces swelling of the joint capsule through an increase in the intra-articular liquid volume following the injection of contrast agents, thus enabling more accurate imaging of intra-articular structures [1]. Intra-articular imaging performed by magnetic resonance (MR) imaging and computed tomography employs various pharmaceutical contrast agents [3]. Iopromide (IPM), a low osmolality non-ionic contrast medium, and gadolinium diethylenetriaminepentaacetate (Gd-DPT), which has a high magnetocaloric effect in acyclic IIIB group are routinely used arthography contrast agents [4–6].

Nevertheless, despite their wide use, previous studies have reported side effects following the use of several contrast agents in clinical settings [7–10]. Various multidisciplinary pharmacogenetic and pharmacogenomic studies have been performed to address damaged tissue repair without causing side/adverse effects [11, 12]. Indeed, recent studies have developed customizable biological treatment models for the regeneration of articular cartilage tissues [10, 13–17].

The purpose of the present study is to assess the possible cytotoxic effects of IPM and Gd-DPT on chondrocytes by blindly comparing the use of these agents in human primer cell cultures at the molecular level in vitro.

## Methods

### Materials

Collagenase type II enzyme (1 mg/mL; Invitrogen Corporation), Hank’s Balanced Salt Solution (HBSS)-1X (Cat. 14025, Gibco), penicillin-streptomycin, fetal calf serum, Dulbecco’s modified Eagle’s medium (DMEM, 1000 mg glucose/L), and an agarose solution (Cat. A9539) used to fix the cells for MR imaging, were all supplied from Sigma Chemicals, USA. Sodium dodecyl sulfate (SDS; cat. L4522), insulin-transferrin-selenous acid premix, and DMEM were supplied from Sigma-Aldrich Gmbh Germany. IPM (300/100 mL) was purchased from Bayer, whereas Gd-DPT was supplied from Schering Corporation. 3-(4,5-dimethylthiazol-2-yl)-2,5-diphenyltetrazolium bromide (MTT) commercial kit (Vybrant MTT cell Proliferation assay, cat. V-13154) was purchased from Cell Biolabs, USA. Stage-specific embryonic antigen-1 (SSEA-1) and the Human Mesenchymal Stem Cell Characterization Kit (Cat. K36094-21A) were obtained from Celprogen, USA.

A laminar current cabinet (cat. NF–800 R) and incubator (cat. 06750) were purchased from Nuve, Turkey. Inverted light microscopy was performed on an Olympus camera (cat. CKX41). The images were evaluated using Olympus Cell Soft Imaging System program. The enzyme-linked immunosorbent assay (ELISA) reader used to measure cytotoxicity and SSEA-1 gene expression was purchased from Mindray MR 96 A, PRC. Environmental scanning electron microscopy (ESEM) was performed on a Quanta 250 FEG (Fei Company, USA). MR was performed on a Siemens Magnetom Skyra 3-tesla (Germany).

### Study design

The researchers were blind to the active ingredient content of the contrast agents added to the cell cultures. In order to minimize bias, all analyses were carried out by the same researcher. All the experiments were performed in triplicate.

Pure human primer chondrocyte cultures were used as the control (group I). Cells in groups II and III were treated with IPM and Gd-DPT, respectively. Finally, group IV cells were treated with Gd-DPT 0.9% physiological saline solution at a 1:250 gadolinium dilution rate, IPM, and a mixed lidocaine solution.

A total of 180 wells were prepared for each group and the five sub-groups, allowing experiments to be performed in triplicate.

Cultures were allowed to progress for 0, 2, and 6 h, at which times the SSEA-1 protein expression, MTT cell viability, toxicity, and proliferation, as well as pre-chondrocyte formation, were compared. Synchronously, the cell surface morphologies of all samples were scanned using inverted light microscopy and ESEM, and with MR imaging at the macroscopic level.

### Eligibility criteria

Osteochondral tissues taken from patients (*n* = 9) surgically treated for knee arthroplasty at the Orthopedics and Traumatology Clinic due to gonarthrosis were included in the study. However, tissues of patients with a hypersensitivity to IPM and Gd-DPT contrast agents (*n* = 1), abnormal thyroid function (*n* = 1), or nephrologic problems (*n* = 1), were excluded from the study. Following the exclusion process, primer chondrocyte cultures were performed in six samples.

### Isolation and cell culture of primary human chondrocytes

**Fig. 1:**
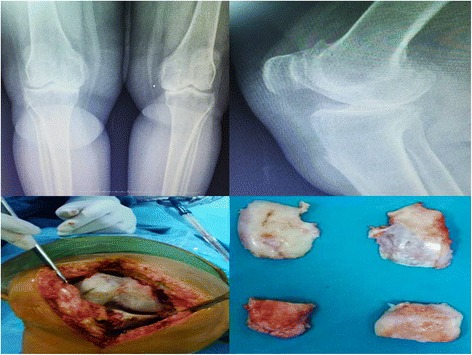
Osteochondral tissue obtained during total knee replacement surgery

Osteochondral tissues from the proximal and distal ends of tibia and femur were resected during total knee arthroplasty. Tissues were transferred to the laboratory using culture medium (Fig. 1) and were placed in laminar cabinets, were washed with 0.9% isotonic sodium chloride solution, and were separated from the red blood cells. Chondral tissues were separated from osteochondral tissues.

Tissue samples were dispersed with a rongeur and were transferred to falcon tubes. Collagenase type II enzyme solubilized in HBSS in accordance with the drug bulletin then added on tissue samples and samples were placed in an incubator overnight at 37.4 °C and a 5% CO_2_ atmosphere. Samples were then centrifuged for 10 min at 120 rpm in a cooled centrifuge. The cell pellet was resuspended in DMEM fresh culture medium, was transferred to a petri dish, and was left for a further 72-h incubation. Following incubation, cells were trypsinized with trypsin-ethylenediaminetetraacetic acid (0.25%). Cells were counted by trypan blue using a Thoma slide, and were placed in 96-well plates at 1.5 × 10^4^ cells/well, in 24-well plates at 3.3 × 10^4^ cells/well, and in 10-mm petri dishes at 4.4 × 10^6^ cells/dish. Cell which were replaced in every 2 days were taken into the incubator for 24 h. With the help of trypsinization and scraper, they were transferred into well plates from petri dishes [1, 8]. Contrast agent addition was commenced as of the second culture passage.

### Preparation and application of drugs in chondrocyte culture samples

Samples in group I was cultured with no contrast agent addition. Main stock solutions were prepared in 50 mL volumes with 0.623 g IPM, 20 mL with 469.01 mg Gd-DPT in each 1 mL aqueous solution, respectively. IPM (15.6 mg/mL) and Gd-DPT (18.7 mg/mL) were applied to the culture samples in groups II and III by dilution with medium. The dose concentrations applied were calculated according to toxicity results from drugs containing similar active ingredients [2].

A mixed contrast agent solution was also prepared by adding 0.8 mL of Gd-DPT solution to 100 mL 0.9% sodium chlorur; 10 mL of this mixture was then mixed with 5 mL IPM and 5 mL lidocaine, diluted by gadolinium at the rate of 1:250. Such a solution is commonly injected into intra-articular joints [2]. Herein, the mixture (18.7 mg/mL) prepared as 1:250 Gd-DPT dilution was added to the wells in group IV (Table 1).

**Table 1:** Agents, commercial stock solution concentrations, and final concentrations

Pharmacological agents	Commercial stock solution concentration (mg/mL)	Final concentration (mg/mL)	Groups
–	–	–	Group I (untreated control group)
Gd-DPT	469.01	18.7	Group II
IPM	623	15.6	Group III
0.9% saline	–	Diluent	Group IV (mixture)
Gd-DPT	469.01	18.7	
IPM	623	15.6	
Lidocaine	100	2.5	

*Gd-DPT* gadolinium-diethylenetriaminepentaacetate, *IPM* iopropamide

Solutions were prepared and stored in light-proof letter coded bottles and delivered to researchers blind to the content of each bottle. The contrast medium was added to the samples, except for the control group, using an automatic pipette with a calculated volume.

### Inverted light microscopy

Micro images of cell organizations belonging to cartilaginous tissue were recorded confocally at ×4, ×10, ×20, and ×40 magnification under phase-contrast microscopy before and after plating in petri dishes. The images were analyzed using Olympus Cell Soft Imaging program.

### ESEM analysis

ESEM analysis was performed to obtain information about surface topography and sample compositions. The cell culture medium and contents were retrieved using a gun pipettor. A cacodylate and glutaraldehyde mixture was used for fixation. The fixation solution was then removed, and samples were left at room temperature for 2 h. Samples were then washed three times with pure cacodylate and were analyzed [10, 13, 15, 18].

FEG ion pumps were used to achieve a high vacuum. The images were recorded at a pressure of 219–231 Pa in ESEM vacuum mode, at ×5000 magnification and 82.9 μm resolution depths (HFW), at an operating voltage of 5.00 kV, and at a working distance of 9.4–10.7 mm.

### MTT-ELISA viability and toxicity proliferation analyses

The viability tests were carried out using an MTT kit (3-[4,5-dimethyltiazol-2-yl]-2,5-diphenyltetrazolium bromide; Thiazolyl blue), which inhibits formazan crystal formation in dead cells [10, 14, 15, 18].

Analyses were performed prior to and at 0, 2, and 6 h following agent addition. The cell culture medium was removed and replaced with a fresh MTT tetrazolium solution (100 μL of stock solution 5 mg–12 mM/6 mL, 1 mL DMEM, and 1 mL sterile PBS; pH = 7.4). A 0.01 M HCl and 1 g/10 mL SDS mixture was also added. Following a 150 min incubation period at 37 °C in a dark environment, 500 μL of medium was removed from the samples. DMSO was then added and the samples were incubated for 10 min at 37 °C. The wave length absorbance was recorded at 540 nm.

By adding 500 μL SDS-HCl solution in the cells left for proliferation tests, they were incubated at 37 °C at 0, 2, 6, and 18 h. Then, absorbances at 570 nm were recorded, thus evaluating cell proliferation [10, 14].

The viability of the control group prior to contrast agent addition was accepted as 100%. Cell viability absorbances were recorded at 2 and 6 h.

### SSEA-1 chondrocytic activity assay

During the differentiation of human mesenchymal stem cells containing embryonic stem cells, SSEA-1 protein expression is upregulated, whereas in cells that do not undergo differentiation expression is downregulated. A pre-chondrocytic human characterization ELISA kit was used to assess whether cells in chondrocyte cultures underwent differentiation, undifferentiation, stimulation, or inhibition by determining changes in SSEA-1 expression in the cultured cells [19-21]. Analyses were performed at 540 nm absorbance in an ELISA reader at 0, 2, and 6 h.

### The evaluation of cell morphology by MR scanning

The samples were prepared through chondrocyte culture at 4.4 × 10^6^ cells/dish in 10-mm petri dishes. At 2 and 6 h following agent application, cells were washed thoroughly with 0.01 M PBS in order to eliminate the volume of agents not taken up by the cells. Cells were covered with 1% agarose gel, which solidified at room temperature, to immobilize them [22].

### MR protocol

All samples were imaged on a 3 T MR scanner. The samples were imaged with T2-weighted haste sequence. The protocol consisted of an spin echo-SE acquisition with a repetition time of 800 ms and echo times of 92 ms. The field of view was 260 × 260 mm, the pixel matrix was 256 × 256 mm, and slice thickness was 2 mm.

### Statistical analyses

Descriptive statistics were shown as mean ± standard deviation. In the analyses of the obtained data, results were evaluated by cell number, proliferation, and SSEA-1 protein expression. The Minitab R16 program was used for statistical evaluation. Evaluations were made at 95% confidence interval.

The results were evaluated using analysis of variance (ANOVA) to assess whether there were significant differences across groups. When differences across groups were observed, Tukey’s honest significant difference (HSD) test, a post-hoc multiple pairwise comparison test, was used to determine the difference and to investigate the false positive, thus evaluating the various averages across experimental groups.

Since there were many measures, and the data were comprised of sub-groups, the Pearson correlation test was used to assess whether there was a direct relation between SSEA-1 and MTT cell proliferation variables.

## Results

### Evaluation through inverted light microscopy and ESEM

**Fig. 2:**
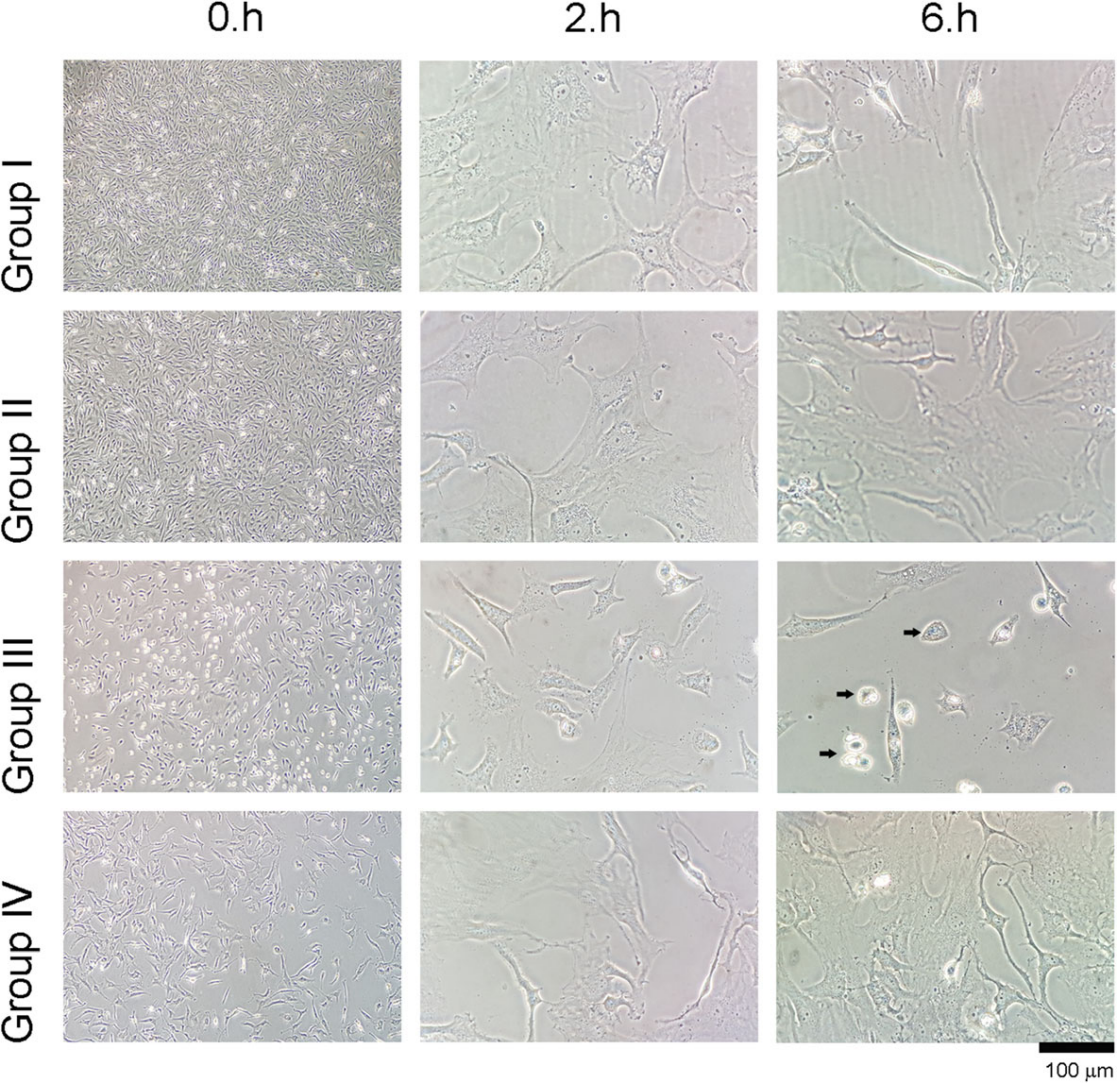
Evaluation of round-shaped chondrocytes through inverted light microscopy

**Fig. 3:**
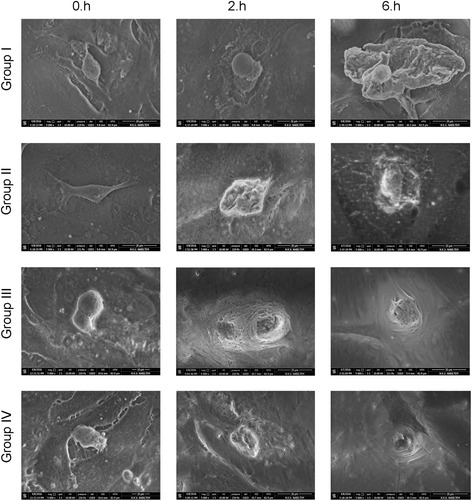
Evaluation of chondrocyte surface morphology through environmental scanning electron microscopy

When the inverted light microscopy and ESEM images were examined, a change in cell morphology was found to be correlated with MTT data (Figs. 2 and 3).

In group III, in which Gd-DPT was used exclusively, shrinkage, typical of a reaction of cells to cytotoxic agents, was observed after 2–6 h. The formation of extracellular matrix was substantially decreased. Chondrocyte cells were detached from the culture vessel and had a round shape, which is an indicator of cytotoxicity.

### Interpretation of 3-tesla MRI

**Fig. 4:**
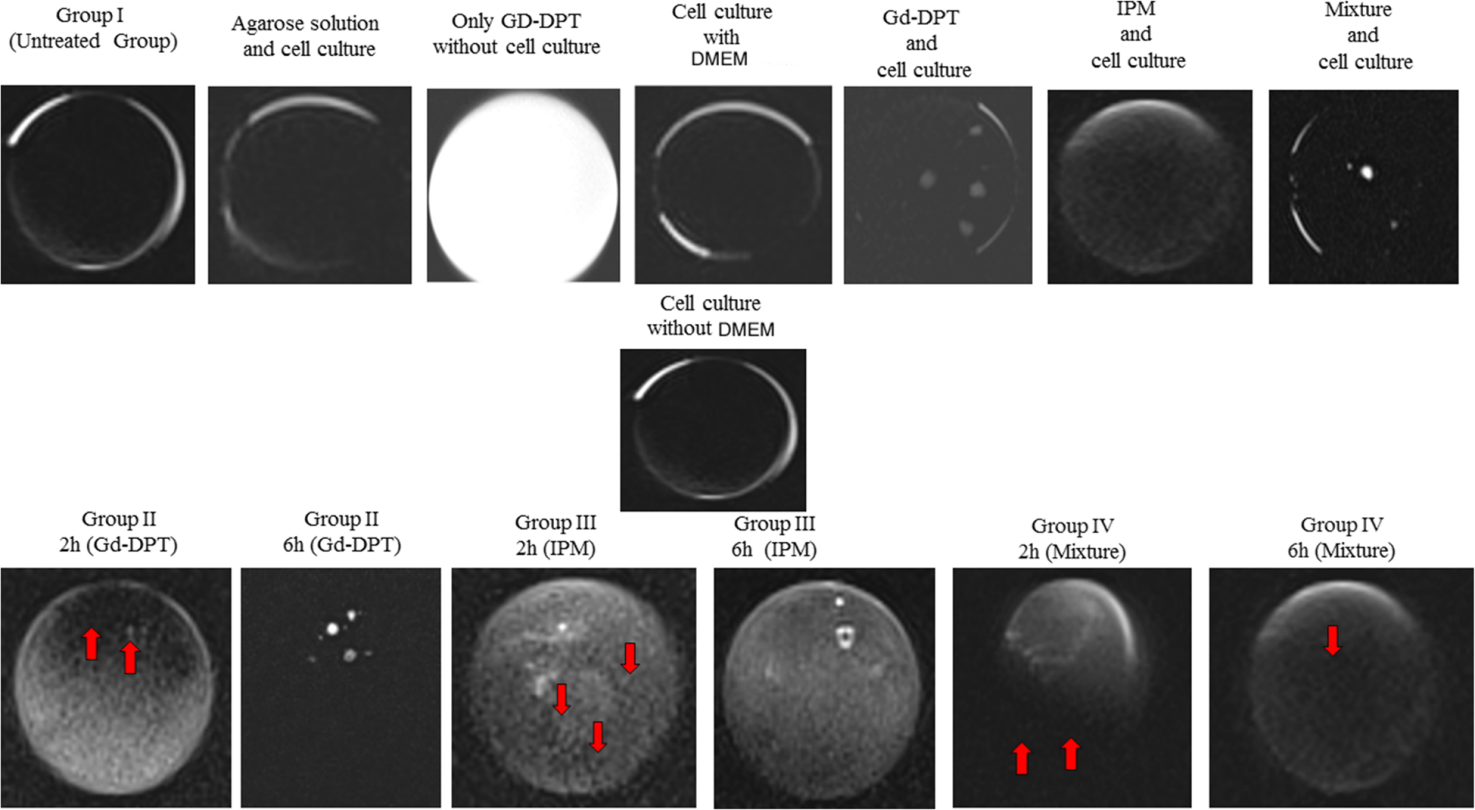
Evaluation of chondrocytes through 3-tesla magnetic resonance imaging

MRI performed in all experimental groups including the control group. Chondrocytes, where Gd-DPT and the mixture solution have been applied, absorbed the contrast agents and monitorized (Fig. 4).

In groups II and III, where IPM and Gd-DPT were applied, loss of cell integrity, and cavity formation was observed after 2 h (red arrow). Considerably more loss of cell integrity and further cell damage was observed after 6 h.

### Statistical analysis of the toxic effects of contrast agents on chondrocytes as MTT-proliferation and SSEA-1 protein expression

The Tukey’s test resulted in a yes/no response to the hypothesis (e.g., are there significant differences between the wells with *P* < 0.05). *P* < 0.01 was considered to be highly significant. Differences across the groups were found through Tukey’s HSD evaluation after ANOVA, and these were statistically significant (Tables 2 and 3).

**Table 2:** Comparison of differences between groups

SSEA-1 versus for analysis of variance					
Source	DF	Adj SS	Adj MS	*F* value	*P* value
Method	11	0.127148	0.011559	505.82	<0.001
Error	60	0.001371	0.000023		
Total	71	0.128519			
MTT-cell proliferation versus for analysis of variance					
Source	DF	Adj SS	Adj MS	*F* value	*P* value
Method	11	0.012400	0.001127	8903.31	<0.001
Error	60	0.000008	0.000023		
Total	71	0.012407	0.000000

**Table 3:** Statistical analyses. Tukey’s pairwise comparisons for SSEA-1 protein and MTT cell proliferation

	*N*	Mean ± St. Dv.	Grouping
Tukey’s pairwise comparisons for SSEA-1 protein (grouping information using the Tukey’s method and 95% confidence interval)			
Group I (control 6 h)	6	0.489500 ± 0.000379	(A)
Group I (control 2 h)	6	0.477067 ± 0.000489	(B)
Group IV (mixture 0 h)	6	0.476467 ± 0.000052	(B)
Group III (Gd-DPT 0 h)	6	0.476450 ± 0.000084	(B)
Group II (IPM 0 h)	6	0.476450 ± 0.000084	(B)
Group I (control 0 h)	6	0.476450 ± 0.000105	(B)
Group II (IPM 2 h)	6	0.464500 ± 0.000000	(C)
Group II (IPM 6 h)	6	0.462500 ± 0.000000	(C)
Group IV (mixture 6 h)	6	0.451667 ± 0.000816	(D)
Group IV (mixture 2 h)	6	0.450167 ± 0.000983	(D)
Group III (Gd-DPT 2 h)	6	0.444133 ± 0.000103	(D)
Group III (Gd-DPT 6 h)	6	0.323167 ± 0.01650	(E)
Tukey’s pairwise comparisons for MTT-cell proliferation (grouping information using the Tukey’s method and 95% confidence interval)			
Group I (control 6 h)	6	0.159017 ± 0.000130	(A)
Group I (control 2 h)	6	0.157450 ± 0.000418	(B)
Group I (control 0 h)	6	0.155900 ± 0.000420	(C)
Group II (IPM 0 h)	6	0.155800 ± 0.000438	(C)
Group III (Gd-DPT 0 h)	6	0.155767 ± 0.000532	(C)
Group IV (mixture 0 h)	6	0.155753 ± 0.000513	(C)
Group III (Gd-DPT 2 h)	6	0.147450 ± 0.000472	(D)
Group II (IPM 2 h)	6	0.141317 ± 0.000293	(E)
Group II (IPM 6 h)	6	0.140817 ± 0.000041	(E)
Group IV (mixture 2 h)	6	0.140667 ± 0.000082	(E) (F)
Group IV (mixture 6 h)	6	0.140117 ± 0.000103	(F)
Group III (Gd-DPT 6 h)	6	0.110333 ± 0.000075	(G)

**Fig. 5:**
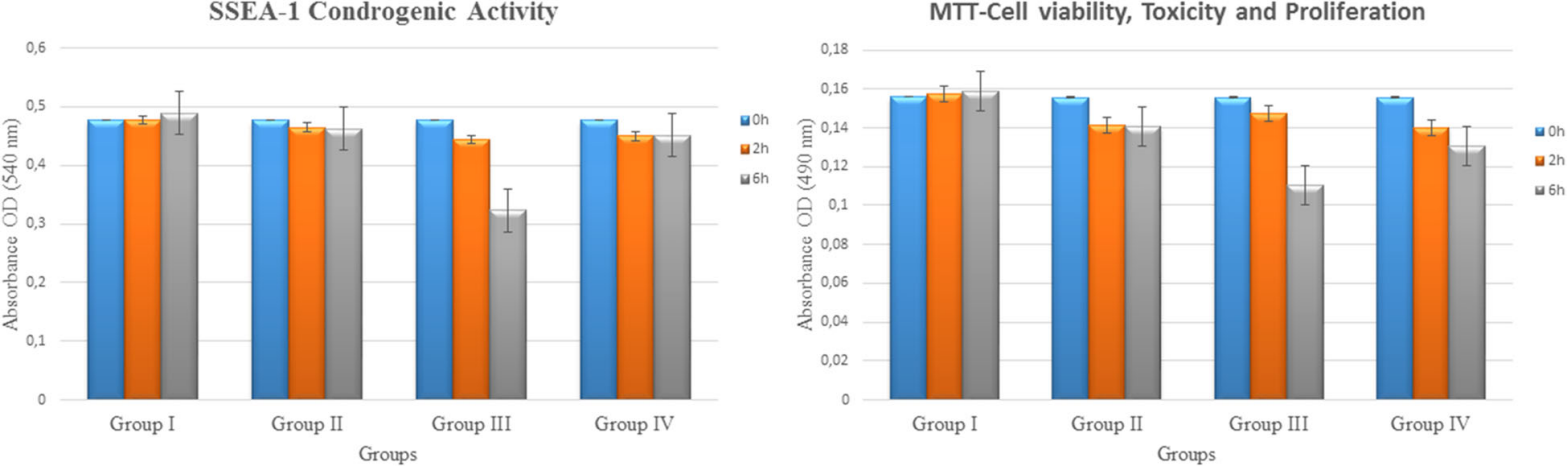
Change of expression pertaining to the SSEA-1 protein component and comparison of the indicators of MTT cell viability, toxicity, and proliferation

When compared with the control group, the lowest chondrogenic differentiation activity was observed in group III at 2 and 6 h, among all agents applied. Cell viability, toxicity, and proliferation were evaluated with MTT analysis and were compared with the non-drug control group. Likewise, the lowest number of cells was observed in group III, where IPM was applied for 6 h (Fig. 5).

SSEA-1 protein expression in a word chondrogenic activity was slightly lower in group IV where a mixed solution was applied than group II, both in 2 and 6 h applications. But decrease in cell viability and proliferation was obvious in group IV at 6 h when compared with group II and control (*P* < 0.001). These differences were statistically significant (Table 2).

A statistically significant correlation between SSEA-1 protein expression and MTT cell viability, toxicity, and proliferation was observed (Pearson correlation sig. (2-tailed); rho = 0.351; *P* = 0.003).

## Discussion

In all areas of medicine, orthopedic surgery addresses the repair of damaged tissues, especially articular cartilage. On the other hand, the protection of healthy tissues is also an important issue [10, 14, 18]. Therefore, studies assessing the toxicity of frequently prescribed drugs on articular cartilage at the molecular level have gained recent popularity [10, 14]. The present study aimed to evaluate the chondrotoxic effects of IPM and Gd-DPT, which are widely applied intra-articular contrast agents in arthrography.

As is well known, destruction in articular cartilage occurs in degenerative diseases such as osteoarthritis, as a result of traumatic, mechanical, genetic, metabolic, and biochemical factors [22]. Further, cartilage tissue and/or cells may be damaged by clinically prescribed pharmaceutical preparations [10, 14, 23–25].

A recent study assessed the cytotoxicity of drugs such as rituximab, adalimumab, abatacept, etanercept, and infliximab in primer cell in vitro cultures isolated from gonarthrosis cases and found that the least damaging biological agents were rituximab and adalimumab, whereas the remaining drugs were seen to negatively affect chondrocytes [10, 26]. A similar study assessed the chondrotoxicity of drugs widely used before and after surgery, such as vancomycin, linezolid, and teicoplanin, was assessed and concluded that these drugs were not chondrotoxic [14]. However, in both studies, the number of cells and proliferation analyses were only statistically evaluated based on mitochondrial activity as from the application of the drugs in a culture environment. A similar in vitro experimental setup was used herein; however, in addition to mitochondrial activity analyses, the proliferation and viability of chondrocytes, the toxicity of IPM, Gd-DPT, and their mixture, and SSEA-1 protein expression were evaluated with spectrophotometer. Thus, whether chondrocyte cells in culture were exposed to differentiation, undifferentitation, stimulation, or inhibition was assessed in addition to an evaluation of drug toxicity and proliferation; these aspects represent the strengths of the present study.

In 2007, the toxicity of iodinated contrast medium was analyzed in various cell cultures in vitro [27]. Further, studies examining the toxicity of contrast substances indicated that ixotitalamate, used in discography or percutaneous endoscopic lumbar discectomy, induced toxicity in the disc nucleus pulposus and not on cartilage cells [28, 29].

A literature search revealed only one study assessing in vitro cell toxicity of cartilage-targeted low-generation dendrimer-linked nitroxide MR contrast agents and gadopentetate dimeglumine using a long-term Swarm rat chondrosarcoma chondrocyte-like cell line [30]. The study evaluated spectrophotometric assays of metabolic activity and cell proliferation and concluded that long-term exposure to either diaminobutyl-linked nitroxides citrate or gadolinium-*d*iethylene*t*riamine*p*ent*a*cetate had no detectable toxicity, with the results being equivalent to untreated cultures.

No studies were retrieved in which the use of IPM contrast agent itself or with Gd-DPT was compared. Further, the limited number of studies in which cartilage tissues or the chondrocyte-directed toxicity were analyzed were carried out on animal models. However, as the physiological structure and/or sensitivity may differ from that of humans, it was reported that the results may not be reliable. In similar studies where animal tissues were not used, commercial cell-lines, known to have lost the in vivo phenotypic and genotypic features, were used instead [10, 14, 18, 21, 28, 31].

In the present study, the cartilage tissue used belonged to patients with knee prosthesis in the course of routine clinical practice. Thus, primer chondrocyte cultures were prepared from undamaged chondral tissues of the resected articular surface, allowing analyses in the natural cell environment, including the extracellular matrix. Further, the present study evaluates both Gd-DPT and IPM contrast agents and the chondrotoxicity of the mixture of these in an in vitro culture environment derived from human tissue.

In control group, regular cell morphology and intact extracellular matrix formation observed with inverted light microscopy. Contrarily, none of the chondrocyte cultures which arthrography agents applied reached confluency. Besides, lose of cell morphology and deterioration of extracellular matrix was observed. Further, ESEM analyses indicated that, in healthy chondrocytes, all natural surface characteristics were visible in the control group, thus supporting inverted light microscopy. Cell viability analysis, quantitative ELISA, and MR imaging also support these morphological data.

Compared with the control group, IPM treatment had the lowest SSEA-1 expression after 6 h of culture followed by the mixed solution and Gd-DPT treatments. The same pattern was observed in the MTT proliferation, cell viability, and toxicity assays–IPM treatment was the most cytotoxic. The differences observed were statistically significant (*P* < 0.001). Further, a statistically significant correlation between SSEA-1 and MTT values and cell proliferation was observed (Pearson product moment correlation sig. (2-tailed); rho = 0.351; *P* = 0.003). These results support those obtained through imaging techniques, whereby round-shaped cells were observed, indicating cytotoxicity. Thus, the present study shows that contrast agents commonly used in intra-articular imaging are toxic to human primer articular chondrocytes, albeit Gd-DPT to lower degree than IPM.

As known, cartilage tissues are avascular and aneuronal and they are deprived of lymph tissues. For this very reason, cartilage cells are fed via synovial liquid which washes articulation surfaces in certain areas without perichondrium or perichondrium layer in vascular shape. Since the outer layer of the synovial fluid is thicker, drugs and/or nutrition diffuse from synovial tissue to the synovial liquid. Afterwards, they pass through pores to the synovial fluid and reach chondrocytes, which results in a second diffusion [32, 33].

It is known that drugs accumulate in the synovial fluid whether they are taken orally or parenterally. Many drugs taken into the body accumulate in the synovial fluid compartment [32, 33].

Even though we carried out our study in primary human chondrocyte cultures but not in the synovial liquid, we doubt that toxification occurs due to accumulation of drugs in the synovial liquid.

We suggest that agents used in this diagnostic methods, which allows for detailed imaging of intra-articular structures, should be administered, if possible, without addition of the local anesthetic agents. It should be remembered that chelated agents when used in combination form may be more harmful to cartilage tissue especially to chondrocytes and extracellular matrix and/or associated tissues when administered intra-articularly in the clinic.

## Conclusions

The present study performed in vitro chondrocyte cultures and compared the detrimental effects of Gd-DPT and/or IPM at low concentrations on cartilage tissue cells and extracellular matrix in terms of cell size in the short term. Further evaluation of Gd-DPT and/or IPM with clinically appropriate long-term exposure times is required to determine the maximum useful concentration. Despite its valuable in vitro results, the present study is limited by its insufficient clinical relevance; therefore, studies evaluating the clinical outcomes in vivo are required.

## Abbreviations

ESEM: Environmental scanning electron microscope
FEG: Field emission guns
HBSS: Hank’s balanced salt solution
HSD: Honest significant difference
MTT: 3-(4,5-dimethylthiazol-2-yl)-2,5-diphenyltetrazolium bromide
NSAIDs: Non-steroidal anti-inflammatory drugs
OD: Optical density
SDS: Sodium dodecyl sulfate
SSEA-1: Stage-specific embryonic antigen-1.7
WD: Wavelength-dispersive
